# Range map data of marine ecosystem structuring species under global climate change

**DOI:** 10.1016/j.dib.2023.110023

**Published:** 2024-01-02

**Authors:** Lidiane Gouvêa, Eliza Fragkopoulou, Térence Legrand, Ester A. Serrão, Jorge Assis

**Affiliations:** a Centre of Marine Sciences, University of Algarve, Faro, Portugal; b Faculty of Bioscience and Aquaculture, Nord Universitet, Bodø, Norway

**Keywords:** Marine ecosystem structuring species, Species distribution modelling, Marine biodiversity, Shared Socioeconomic Pathway (SSP) scenarios, Range maps

## Abstract

Data on contemporary and future geographical distributions of marine species are crucial for guiding conservation and management policies in face of climate change. However, available distributional patterns have overlooked key ecosystem structuring species, despite their numerous ecological and socioeconomic services. Future range estimates are mostly available for few species at regional scales, and often rely on the outdated Representative Concentration Pathway scenarios of climate change, hindering global biodiversity estimates within the framework of current international climate policies.

Here, we provide range maps for 980 marine structuring species of seagrasses, kelps, fucoids, and cold-water corals under present-day conditions (from 2010 to 2020) and future scenarios (from 2090 to 2100) spanning from low carbon emission scenarios aligned with the goals of the Paris Agreement (Shared Socioeconomic Pathway 1-1.9), to higher emissions under reduced mitigation strategies (SSP3-7.0 and SSP5-8.5). These models were developed using state-of-the-art and advanced machine learning algorithms linking the most comprehensive and quality-controlled datasets of occurrence records with high-resolution, biologically relevant predictor variables. By integrating the best aspects of species distribution modelling over key ecosystem structuring species, our datasets hold the potential to enhance the ability to inform strategic and effective conservation policy, ultimately supporting the resilience of ocean ecosystems.

Specifications Table

**Table:** 

Subject	Ecology, Biodiversity, Climate Change.
Specific subject area	Species distribution modelling, Biodiversity information, Marine biogeography, Marine conservation and management.
Data format	Raw data in Excel files Raster layers as GeoTIFF
Type of data	Tables, Figures and Range maps
Data collection	Georeferenced occurrence records of marine ecosystem structuring species (i.e., seagrasses, kelps, fucoids, and cold-water corals) were accessed from expert-curated datasets that aggregate data from biodiversity information facilities and peer-reviewed scientific literature. Environmental data were accessed from Bio-ORACLE for present-day and contrasting end-of-century climate change conditions.
Data source location	Occurrence records of marine ecosystem structuring were gathered from: 1. A fine-tuned dataset of marine forests (i.e., seagrasses, kelps, fucoids) at global scale. 2. A dataset of cold-water coral distribution records. 3. Bio-ORACLE v3.0, pushing marine data layers to the next-generation scenarios of climate change research. Institution: CCMAR, Centre of Marine Sciences, University of Algarve, Faro, Portugal.
Data accessibility	Repository name: Figshare (Sups. 1-3) and GitHub (Sup. 4) Data identification number: Direct URL to data: https://doi.org/10.6084/m9.figshare.23749179 and https://github.com/jorgeassis/speciesDistributionModelling Supplement 1. Occurrence records and environmental data used in species distribution modelling of marine ecosystem structuring species; Supplement 2. Performance of species distribution modelling of marine ecosystem structuring species, relative contribution (%) and tipping points of predictors variables. Supplement 3. Range maps and uncertainty maps per species under present-day conditions and future climate change scenarios. Supplement 4. R code used to develop species distribution modelling

## 1 Value of the Data

• Range maps of marine species were built with machine learning modelling fitting biodiversity data and relevant predictor variables under present-day conditions and future scenarios of climate change.
• A new baseline to estimate present-day biogeographic patterns, explore niche-based questions and phylogeographic hypotheses.
• Important information at the global scale to explore the potential impacts of future climate change to guide conservation, management, and restoration actions.

## 2 Background

We provide range maps of marine structuring species (i.e., seagrasses, kelp forests, fucoids, and cold-water corals) for present-day and future climate change scenarios, spanning from low carbon emissions aligned with the goals of the Paris Agreement, to high emissions under reduced mitigation strategies, specifically the SSP1-1.9, SSP3-7.0 and SSP5-8.5 of the next generation CMIP version 6. The range maps were developed using an ensemble of machine learning Species Distribution Modelling (SDM) combining comprehensive datasets of occurrence records with high-resolution, biologically relevant environmental predictor variables. The datasets are available under the FAIR principle of Findability, Accessibility, Interoperability and Reusability.

## 3 Data Description

The dataset was generated using machine learning SDM for 980 marine ecosystem structuring species of seagrasses, kelp forests, fucoids and cold-water corals. SDMs are statistical tools that allow linking environmental predictor variables with occurrence records to estimate species distribution at the global scale [1]. Specifically, we produced predictive habitat suitable maps per species [2] under present-day conditions and future scenarios of climate change at global scale, as well as uncertainty maps depicting the standard deviation of predictive responses.

Moreover, we assessed the predictive performance of the models under a cross-validation framework, determined the relative contribution of each predictor to the distribution of each species and identified hypothetical physiological tolerance limits (tipping points) for each predictor variable [[3], [4]].

The models utilized 2,751,458 occurrence records derived from datasets that provide expert-revised biodiversity data [[5], [6], [7]] (Supplement 1) [8]. It specifically concerns 1,048,576 occurrence records of 59 seagrass species, 629,491 records of 103 kelp forest species, 377,986 records of 239 fucoid species, and 695,405 records of 579 cold-water corals species. Tables S1-S8 available in Excel format, provide additional information (Supplement 1) [8]. The models employed relevant environmental predictor variables for present-day and future climate change scenarios (Table 1; Supplement 1) extracted from the Bio-ORACLE dataset v3.0 [9]. These predictors included maximum and minimum ocean temperature, sea ice cover, nitrate, salinity, pH, total phytoplankton, dissolved molecular oxygen, seawater velocity, topographic slope, terrain ruggedness index and wave energy. All predictor variables are available as GeoTIFF raster layers (Supplement 1) [8].

**Table 1:** Predictor variables used in species distribution modelling. Predictor variable, unit, sea depth, marine group, period, description, and file name are reported (Raster files are present in Supplement 1).

Predictor	Unit	Group	Period	Description	File name
Ocean temperature	°C	Seagrasses, kelps, fucoids and cold-water corals	Present-day and future	Long-term average of monthly maximum	OceanTemperature BenthicMean LtMax.tif OceanTemperature BenthicMin Ltmax.tif
Ocean temperature	°C	Seagrasses, kelps, fucoids and cold-water corals	Present-day and future	Long-term average of monthly minimum	OceanTemperature BenthicMean LtMin.tif OceanTemperature BenthicMin LtMin.tif
Sea ice cover	Fraction	Seagrasses, kelps, fucoids	Present-day and future	Long-term average of monthly minimum	SeaIceCover Surface Ltmin.tif
Nitrate	mol.m^−3^	Seagrasses, kelps, fucoids	Present-day and future	Long-term average of monthly minimum	Nitrate BenthicMin Ltmin.tif
Salinity	-	Seagrasses, kelps, fucoids and cold-water corals	Present-day and future	Long-term average of monthly minimum	Salinity BenthicMean Ltmin.tif Salinity BenthicMin Ltmin.tif
pH	-	Cold-water corals	Present-day and future	Long-term average of monthly minimum	pH BenthicMean Ltmin
Total phytoplankton	μmol . m^−3^	Cold-water corals	Present-day and future	Long-term average of monthly minimum	TotalPhytoplankton BenthicMean Ltmin
Dissolved molecular Oxygen	mol.m^−3^	Cold-water corals	Present-day and future	Long-term average of monthly minimum	DissolvedMolecularOxygen BenthicMean Ltmin
Sea Water Speed	m.s^−1^	Cold-water corals	Present-day and future	Long-term average of monthly minimum	SeaWaterSpeed BenthicMean Ltmin.tif
Topographic Slope	-	Cold-water corals	Present-day	-	Slope BenthicMean.tif
Terrain Ruggedness index	-	Cold-water corals	Present-day	-	TerrainRuggednessIndex BenthicMean.tif
Wave energy	-	Seagrasses, kelps, fucoids	Present-day	-	Waveenergy.tif

**Fig. 1:**
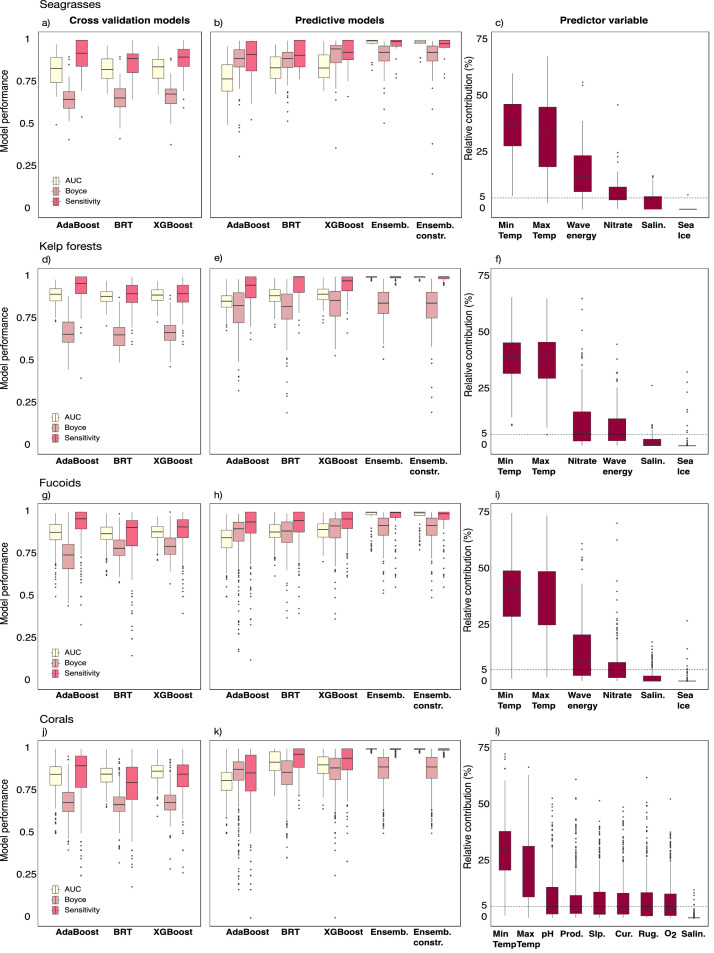
Performance of Species Distribution Modelling inferred with (a) cross-validation and (b) the final predictive models based on Adaptive Boosting (AdaBoost), Boosted Regression Trees (BRT), Extreme gradient boosting (XGBoost), and the ensemble of algorithms (without and with dispersal constraints), estimated with AUC, Boyce, and Sensitivity (yellow, light pink, and pink, respectively). (c) The relative contribution of each predictor variable to the ensemble of the algorithms (for more information, refer to Supplement 2, Tables S11-S22).

The SDM considered three high-performance machine learning algorithms: Adaptive Boosting (AdaBoost) [10], Boosted Regression Trees (BRT) [[10], [11]] and Extreme Gradient Boosting (XGBoost) [12] (Figure 1). The performance of each modelling algorithm, as well as the performance of their ensemble (i.e., weighted averaged ensemble modelling) [13], were determined by parameters like the Boyce index, the area under the receiver operating characteristic curve (AUC), and sensitivity [3] under a cross-validation framework and for the final predictions. The relative contribution (%) of each variable predictor was further determined to assess the significance of the models. For further details, please refer to Supplement 2 [8].

The data sources and range maps of marine ecosystem structuring species are publicly available in a permanent repository (Figshare at https://doi.org/10.6084/m9.figshare.23749179) [8] containing the following files:

(1) Supplement 1: Occurrence records and environmental data used in species distribution modelling of marine ecosystem structuring species (Excel files and Raster layers as GeoTiff).
(2) Supplement 2: Performance of species distribution modelling of marine ecosystem structuring species, the relative contribution of predictor variables (%) and tipping points of predictors variables (Excel files).
(3) Supplement 3: Range maps and uncertainty maps per species under present-day conditions and future climate change scenarios (Raster layers as GeoTiff).

A comprehensive overview of these files is provided in Table 2.

**Table 2:** An overview of supplementary information available in Figshare at https://doi.org/10.6084/m9.figshare.23749179. Folder title and respective content are reported.

**Folder title**					**Content**
**Supplement 1**	**Data**		Climate	Baseline SSP1-1.9 SSP3-7.0 SSP5-8.5	The environmental data are available for present-day conditions, referred as Baseline, as well as for future climate change scenarios, specifically SSP1-1.9, SSP3-7.0, and SSP5-8.5. These folders comprise 17 predictor variables.
			Occurrence records		The occurrence records for each group are listed in Tables S1, S3, S5 and S7. These tables contain two columns representing geographic coordinates (Lon, Lat) in decimal degrees of each species in rows. The WORMS list (World Register of Marine Species) with accepted taxonomies are provided in Tables S2, S4, S6 and S8.
**Supplement 2**	**Statistics**	Supplement 2A-Seagrasses; Supplement 2B-Fucoids; Supplement 2C-Kelps; Supplement 2D-Cold-water corals;	Performance		The performance of models is reported in Tables S11, S14, S17 and S20 organized by species and considering cross-validation, predictive models and the ensemble of the algorithms. The columns refer to AUC, sensitivity, specificity, Boyce and TSS values according to algorithm (BRT, AdaBoost and XGBoost).
			Relative contribution		In Tables S12, S15, S18 and S21, the relative contribution of predictors (%) for each species is displayed in the rows, as calculated by the algorithms (BRT, AdaBoost, and XGBoost) and their ensemble.
			Tipping points		The tipping points of each predictor variable resulting from partial plots are reported for each species in rows. These data are derived from the ensemble of three algorithms and can be found in Tables S13, S16, S19 and S22.
**Supplement 3**	**Range maps**		Seagrasses, kelps, fucoids and corals		Each folder within the repository contains accessible range maps, accompanied by their respective uncertainties for each model. The range maps for present-day conditions are labeled as "Baseline," while those representing future climate change scenarios are designated "ssp." In total, there are 19,716 range maps available, distributed as follows: 708 for seagrasses, 1,308 for kelps, 2,880 for fucoids and 7,032 for cold-water corals.

## 4 Experimental Design, Materials and Methods

### 4.1 Occurrence data

Occurrence records of marine ecosystem structuring species were collated from published datasets for seagrasses, kelps and fucoids [[5], [7]] and cold-water corals [6] (Supplement 1; Tables S1, S3, S5 and S7). Taxonomy was verified for each species using the World Register of Marine Species (WoRMS) (Supplement 1, Tables S2, S4, S6 and S8) [8].

### 4.2 Environmental data

Environmental data for modelling were downloaded from Bio-ORACLE v3.0 [9] at a 0.05° resolution (approx. 5 km at the equator) for present-day conditions (decade 2010–2020) and the future (decade 2090–2100) under three distinct Shared Socioeconomic Pathway (SSP) scenarios: (1) SSP1-1.9, which aims to keep greenhouse gas emissions at a very low level, with a focus on limiting global warming to 1.5 °C above pre-industrial levels; (2) SSP3-7.0, characterized by high greenhouse gas emissions, leading to a projected increase in CO_2_ levels, approximately doubling from current levels by the year 2100 and (3) SSP5-8.5, an extremely high greenhouse gas emission pathway scenario, with CO_2_ levels expected to roughly double from current levels by 2050. Predictor variables were chosen based on the biological relevance of each group considered (Table 1; Supplement 1) [8]. The selection of a subset of relevant predictors was carefully designed to achieve parsimony while increasing the temporal transferability of the models [[14], [15]].

### 4.3 Modelling

We used three machine learning algorithms, namely Adaptive Boosting (AdaBoost) [10], Boosted Regression Trees (BRT) [[10], [11]], and Extreme Gradient Boosting (XGBoost) [12]. These are known to have high performance and the ability to capture complex interactions between predictor and response variables. Furthermore, these statistical tools are able to cope with limited data [16] and allow tuning hyperparameters in order to reduce overfitting and improve model transferability [11].

Since the models are based on species occurrence records, pseudo-absences were randomly generated in regions where no occurrences were reported. In this step, a filtering process was applied to occurrences and pseudo-absences to reduce the potential effect of spatial autocorrelation and sampling bias in distribution models [17]. This involved randomly selecting one record from the pool of occurrences within the minimum distance showing significant spatial autocorrelation [[18], [19]]. To estimate this distance, Pearson's correlation coefficients among predictor variables were evaluated as functions of geographical distance [20]. The number of pseudo-absences was balanced to a 1:1 ratio with occurrence records [16] for species that had more than 1,000 occurrences. For species with fewer occurrences, 10 model runs were performed, each involving a minimum of 100 pseudo-absences, according to [16]. Furthermore, to reduce the likelihood of generating redundant information for modelling, pseudo-absences were climatically structured by applying to each one a unique membership attributed by K-means clustering performed on the predictors and setting the k parameter to the desired number of pseudo-absences [21]. This step further allowed removing the potential negative effect of class imbalance, which is particularly important for machine learning algorithms, and provided a straightforward approach to isolate the potential contribution of predictor variables [21].

#### 4.3.1 Cross-validation of models

To evaluate model performance and predictive error (Supplement 2) [8], we implemented a 10-fold cross-validation framework [[22], [23]] using spatially independent hexagons with sizes corresponding to the previously determined uncorrelated distance [[23], [24]]. Using the cross-validation step, the optimal combination of hyperparameters for AdaBoost, BRT, and XGBoost was identified [11]. This process involved training competitive models on nine randomly selected data folds, while one-fold was reserved for testing the performance in each run. The procedure was performed using the grid search method by testing a span of learning rate (0.1, 0.01 and 0.001), tree complexity (1–4), and number of trees (50–1000, step 50) for BRT, number of interactions (50–250, step 50), degrees of freedom (1–12) and shrinkage (0.25–1, step 0.25) for AdaBoost, and gamma (0–5, step 1), interaction depth (1–4), shrinkage (0.1–0.5, step 0.1) and number of rounds (10–100, step 10) for XGBoost. Monotonic responses [4] were positively or negatively forced to reduce overfitting according to expected outcomes on species distribution. Maximum temperature, sea ice cover, wave energy and sea water speed were hypothesized to have a negative effect on species. In contrast, minimum temperatures, salinity, nutrients, pH, O_2,_ productivity, terrain slope, ruggedness and silicate were assumed to influence species occurrence positively. Pearson's correlation coefficient (r) and Variance Inflation Factor (VIF) were calculated among predictor pairs.

#### 4.3.2 Model evaluation

We evaluated the performance of SDMs using the Boyce index, which is a proper metric for presence-only models [25], as well as with the area under the receiver operating characteristic curve (AUC) and sensitivity [3]. The Boyce index ranges from −1 to +1, while AUC and TSS (true skill statistics) are between 0 and 1. Positive Boyce index values above 0, or AUC and TSS above 0.5, indicate model predictions outperform random expectations, while values neighboring to 1 suggest strong agreement between the model's predictions and the observed patterns [26]. Full models, incorporating all predictor variables, were constructed for each species and algorithm using the combination of hyperparameters retrieving higher performance in cross-validation (Tables S11, S14, S17 and S20) [8]. The significance of these models was assessed by analyzing the relative contribution of predictors to the model's performance (Tables S12, S15, S18 and S21) [8]. Additionally, partial dependence plots were developed, allowing the extraction of hypothetical physiological tolerance limits, either minimum or maximum, depending on the predictor [[11], [27]] (Supplement 2, Tables S13, S16, S19 and S22) [8].

Maps of habitat suitability for individual species were produced for present-day conditions and the SSP scenarios by ensembling the responses of the three algorithms (i.e., weighted averaged ensemble modelling [13] (Supplement 3) [8]. These were then reclassified into binomial maps (Supplement 3) [8] to represent presences and absences, using the minimum training area threshold, which sets the minimum predicted area while keeping sensitivity higher or equal to 0.95 [28]. To reduce overprediction, maps were clipped by accounting for potential reachable areas through dispersal [29], [30], [31], [32], a crucial step when analyzing species with low dispersal ability. This approach considered a fixed maximum dispersal distance of 200 km [33] under the assumption that while dispersing, propagules cannot transpose geographic regions of unsuitable habitat conditions except when demonstrated by occurrence records [32].

#### 4.3.3 Limitations

Projecting climate change impacts on seagrass distributions in regions where future conditions may be different from those experienced by species anywhere in the present day could introduce uncertainties in the models [34]. Additionally, projections do not incorporate additional drivers, such as anthropogenic impacts (e.g., degradation, pollution) [35] or biotic interactions between species (e.g., competition, commensalism) that can influence the distribution of species across space and time [[14], [36]]. Unfortunately, the unavailability of such data at the global scale poses a current limitation. Additionally, the lack of information on the available substrata (e.g., rock bottoms for marine forests and corals), as well as future light conditions, could have resulted in overpredicting suitable habitats [10]. To deal with this limitation, the predicted distribution of species was restricted to their maximum known (i.e., reported) depth. However, these conditions might undergo alterations in the future, particularly in higher latitudes, due to the melting of glaciers and an increase in river outflow. Potential consequences of future sea-level rise altering coastlines were also not considered but could further influence individual assessments of suitable habitats [37].

## Ethics Statements

The present work did not involve human subjects, animal experiments, or any data collected from social media platforms.

## CRediT authorship contribution statement

**Lidiane Gouvêa:** Conceptualization, Data curation, Writing – review & editing. **Eliza Fragkopoulou:** Writing – review & editing. **Térence Legrand:** Writing – review & editing. **Ester A. Serrão:** Funding acquisition, Writing – review & editing. **Jorge Assis:** Funding acquisition, Conceptualization, Data curation, Writing – review & editing, Supervision.

## Data Availability

Range map data of marine ecosystem structuring species (Original data) (Figshare)
